# The quality of life and associated factors in patients on maintenance hemodialysis – a multicenter study in Shanxi province

**DOI:** 10.1080/0886022X.2017.1398095

**Published:** 2017-11-15

**Authors:** Xiaoshuang Zhou, Fuping Xue, Hui Wang, Yufeng Qiao, Guangzhen Liu, Liping Huang, Deqian Li, Shuanggui Wang, Qunyuan Wang, Larong Li, Rongshan Li

**Affiliations:** a Department of Nephrology, Shanxi Provincial People’s Hospital, Taiyuan, Shanxi, China;; b Department of Nephrology, Shanxi Provincial Corps Hospital of Chinese People’s Armed Police Forces, Taiyuan, Shanxi, China;; c Department of Nephrology, Taiyuan Central Hospital, Taiyuan, Shanxi, China;; d Department of Nephrology, Second Hospital of Shanxi Medical University, Taiyuan, Shanxi, China;; e Department of Nephrology, Shanxi Traditional Chinese Medical Hospital, Taiyuan, Shanxi, China;; f Department of Nephrology, The Second Shanxi Provincial People’s Hospital, Taiyuan, Shanxi, China;; g Department of Nephrology, General Hospital for Workers of Xi Shan Coal and Electricity Group, Taiyuan, Shanxi, China;; h Department of Nephrology, PLA 264th Hospital, Taiyuan, Shanxi, China;; i Department of Nephrology, Xiangfen County People's Hospital, Xiangfen, Shanxi, China

**Keywords:** End-stage renal disease, blood purifying center, hemodialysis, quality of life, SF-36 scale

## Abstract

**Objectives:** To assess the quality of life (QOL) and factors affecting QOL in hemodialysis patients so as to improve QOL of dialysis patients and provide the basis for better clinical care.

**Methods:** A retrospective study was performed to assess the QOL and factors affecting QOL in hemodialysis patients. We recruited 125 patients who had been receiving hemodialysis for at least 2 years in the dialysis units of nine hospitals in Shanxi Province, China, and conducted a multi-center questionnaire survey between 1 May 2015 and 1 July 2016. We investigated the patients’ general condition and clinical data and used the Short Form-36 (SF-36) scale to measure QOL in these patients.

**Results:** The overall SF-36 score was 107.55 ± 14.50 in patients who had received hemodialysis for more than 2 years. Age (*p <* .05, *F =* 4.972) and gender (*p <* .01, *t =* 3.993) significantly affected the overall QOL score in these patients. Education level was also an influencing factor (*p <* .05, *Z*= −0.838), especially on the mental health of these patients. In addition, residual urine volume (*p <* .05, *Z*= −2.465) and diabetic nephropathy (*p <* .05, *Z*= −2.062) were important factors that affected the physical strength and QOL score in these patients. However, sources of medical expenses, marital status and different methods of dialysis, had no effect on the QOL score.

**Conclusion:** The overall score of QOL in patients who have received maintenance hemodialysis for more than 2 years is higher in Shanxi Province than that in other provinces of China. Only a few factors influenced the QOL of these patients.

## Instruction

As the major substitutive therapy for end-stage renal disease (ESRD), hemodialysis is the main approach used to cure chronic renal failure (CRF) in an integrated manner. Due to the gradual increase in patients with chronic kidney diseases, the number of patients requiring dialysis is also increasing annually. Nowadays, doctors involved in dialysis treatment not only focus on the therapeutic effects, but also attach importance to the patients’ psychological health, that is improvement in survival quality. Therefore, it is critical to improve the quality of life (QOL) of patients receiving dialysis. Research on QOL began in Western countries, thus evidence on QOL has accumulated over the years. In contrast, this issue has been ignored for a long time in China. In recent years, more emphasis has been placed on QOL and it has become a hot issue in clinical work. To date, there have been many analyses and studies on the QOL of patients receiving dialysis, but reports on this issue in Shanxi Province are scarce. In this study, QOL and the factors influencing QOL were investigated in 125 patients who had received hemodialysis for more than 2 years in Shanxi Province, China. The study was conducted in order to improve the patients’ survival quality, enhance their confidence in treatment and provide assistance to patients undergoing integrated therapy.

## Methods

### Respondents

In this study, 187 volunteers were selected from a sample of 125 patients receiving dialysis for more than 2 years in nine blood purifying centers in Shanxi Province, China (Shanxi Provincial People’s Hospital, Shanxi Provincial Corps Hospital of Chinese People’s Armed Police Forces, Taiyuan Central Hospital, Second Hospital of Shanxi Medical University, Shanxi Traditional Chinese Medical Hospital, The Second Shanxi Provincial People’s Hospital, General Hospital for Workers of Xi Shan Coal and Electricity Group, PLA 264th Hospital and Xiangfen County People's Hospital). The inclusion criteria were as follows: the time of maintenance hemodialysis must be at least 2 years; the patients must be at least 18 years old; the patients must not have serious cardiac, cerebral or pulmonary complications; and the patients must have the ability to listen, speak and respond, and be willing to participate in this investigation. Patients with cardiac, cerebral or/and pulmonary complications, and those unable to complete the questionnaire were excluded from the investigation.

### Methodology

In this study, a questionnaire was adopted to obtain patient data. The questionnaire consisted of three parts. The first part was designed to collect general information, including gender, age, nationality, educational background, source of funding for dialysis and marital status. The second part was designed to collect clinical data, including residual urine volume, primary illness and form of dialysis. The final part was completion of the SF-36 Questionnaire. All questions were answered by the patients under the guidance of specially assigned persons, and the questions involving specialized knowledge which the patients did not understand were completed with the assistance of the hospital doctors where the patients were receiving dialysis. The SF-36 Scale includes 36 questions classified into eight dimensions, which are physiological function (PF), body pain (BP), general health (GH), vitality, social function (SF), role-emotion (RE) and mental health (MH). The eight dimensions can be divided into two distinct, summary scores: physical component summary (PCS) and mental component summary (MCS). The scoring and statistics of each dimension were completed using a specified method as described previously [1].

### Statistical analysis

All data were statistically analyzed using the SPSS13.0 statistical package (SPSS software version 13.0, SPSS Inc., Chicago, IL). The quantitative data were denoted by x¯±s. The data conforming to normal distribution and homogeneity of variance were analyzed using the *t*-test; those not conforming to normal distribution and homogeneity of variance were analyzed using a nonparametric test. The categorical data were compared using the χ^2^ test. The scoring variances of each dimension in blood dialysis patients were compared by *t* test, single-factor variance analysis and the rank sum test. *p <* .05 was considered statistically significant.

## Results

### Data on hemodialysis patients

As shown in Table 1, a total of 125 patients were included in this investigation. These patients had received maintenance hemodialysis for more than 2 years at nine renal care centers in Shanxi Province. Of the selected patients, 65 were male and 60 female; 13 were 35 years old or less, 28 were 36–50 years old, 39 were 51–65 years old, and 45 were 66 years old or older; 60 had received higher education; 21 cases were treated at their own expense and 104 had health insurance; 17 cases were single and the remaining 108 were married; 122 cases were Han and the remaining three were from other nationalities; 106 cases had residual urine volume and the remaining 19 did not; the primary illness was diabetes in 34 cases, while the remaining 99 received simple hemodialysis.

**Table 1.** Baseline characteristics of hemodialysis patients (*n* = 125).

	Number (*n*)	Percentage (%)
Gender		
Male	65	52.0
Female	60	48.0
Age		
≤35	13	10.4
36–50	28	22.4
51–65	39	31.2
≥66	45	36.0
Education levels		
With higher education	60	48.0
Without higher education	65	52.0
Medical insurance		
Personal expense	21	16.8
Health insurance	104	83.2
Marriage		
Single	17	13.6
Married	108	86.4
Nationality		
Han	122	97.6
Others	3	2.4
Residual renal function		
With	106	84.8
Without	19	15.2
Primary disease		
Diabetes	34	27.2
Simple hemodialysis		
Hemodialysis only	99	79.2

### QOL score in hemodialysis patients and influencing factors

*QOL scoring with SF-36.* The total QOL score in the patients in this study as shown by the SF-36 scale was 107.55 ± 14.50. The score for each dimension of QOL is listed in Table 2.

**Table 2.** Scores for each dimension of QOL in hemodialysis patients.

Influencing factors	Score
Physical component scores (PCS)	
Physiological function	65 ± 28
Physical role	67 ± 46
Body pain	85 ± 20
General condition	53 ± 16
Mental component scores (MCS)	
Vitality	60 ± 14
Social function	62 ± 22
Role-emotion	82 ± 38
Mental health	73 ± 11
Total score	107.55 ± 14.50

*Impact of gender on QOL.* The total score in female patients was significantly lower than that in male patients (*p < *.01). In the physiological function (PF), physical role (PR) and body pain (BP) dimensions, the score in male patients was significantly higher than that in female patients. In other dimensions, there were no statistically significant differences between males and females **(**Table 3**)**.

**Table 3.** Total score and score for each dimension in hemodialysis patientswith regard to gender.

	Male (65)	Female (60)	*t v*alue	*p v*alue
PCS				
PF	74.08 ± 21.25	56.08 ± 30.48	3.853	**.010**
PR	79.62 ± 38.50	55.00 ± 50.17	3.091	**.000**
BP	88.82 ± 17.52	80.03 ± 22.40	2.453	**.039**
GC	56.78 ± 13.91	49.38 ± 17.24	2.651	.058
MCS				
VT	64.46 ± 11.73	55.00 ± 15.38	3.885	.197
SF	65.58 ± 20.61	58.96 ± 22.68	1.709	.208
RE	84.62 ± 34.90	78.89 ± 40.69	0.846	.062
MH	73.35 ± 1.84	72.33 ± 11.10	0.496	.814
Total score	112.25 ± 11.75	102.46 ± 15.53	3.993	**.003**

BP: body pain; GC: general condition; MCS: Mental Component Scores; MH: mental health; PCS: Physical Component Scores; PF: physiological function; PR: physical role; RE: role-emotion; SF: social function; VT: vitality.

*p <* .05 indicates statistical difference between male and female (marked in bold).

*Impact of age on QOL.* As shown in Table 4, the total QOL score decreased with increasing age, and the differences were statistically significant (*p < *.05). The total score for the PF, vitality and SF dimensions decreased gradually with increasing age, and the differences were statistically significant (*p < *.05). In other dimensions, the scores also decreased with increasing age, but the differences were not significantly different.

**Table 4.** Total score and score for each dimension in hemodialysis patients with regard to age.

	≤35	36–50	51–65	≥66	*t*	*p*
PCS						
PF	84.62 ± 8.28	77.50 ± 28.04	68.46 ± 26.06	49.78 ± 24.19	11.103	**.000**
PR	82.69 ± 32.89	78.57 ± 41.78	62.82 ± 48.28	61.11 ± 48.72	1.452	.231
BP	87.46 ± 20.41	89.02 ± 17.82	81.59 ± 23.60	83.64 ± 19.00	0.835	.477
GC	54.46 ± 14.54	58.75 ± 14.85	51.67 ± 18.47	50.80 ± 14.25	1.638	.184
MCS						
VT	65.38 ± 7.76	64.81 ± 13.44	58.59 ± 16.22	56.44 ± 13.72	2.827	**.041**
SF	65.38 ± 17.04	71.88 ± 24.21	60.90 ± 20.50	56.94 ± 18.18	2.983	**.034**
RE	74.36 ± 43.36	88.10 ± 31.71	74.36 ± 42.90	86.67 ± 34.38	1.188	**.034**
MH	70.15 ± 13.62	75.14 ± 10.23	72.20 ± 12.07	72.80 ± 11.11	0.650	.585
Total score	112.95 ± 9.64	114.44 ± 13.48	106.57 ± 15.51	102.57 ± 14.50	4.972	**.030**

*p* < .05 indicates statistical difference between age groups (marked in bold).

*Influence of other factors on QOL.* Educational background, source of funding and marital status did not significantly influence QOL. However, compared with other dimensions, educational background had a slightly greater influence on QOL: patients who had graduated from junior colleges or higher educational institutes (73.52 ± 10.26) had higher scores than those whose educational background was lower than junior college (68.71 ± 17.10). The difference was statistically significant (*p < *.05). There were no significant differences in the other dimensions.

*Influence of patients’ clinical data on QOL.* Residual urine volume, the primary causes (such as diabetes) of uremia, and the dialysis method did not significantly influence the total QOL score. In comparison of the scores for all dimensions, the scores in patients with residual urine volume were greater than those in patients with no residual urine volume (66.84 ± 10.96 vs. 58.68 ± 14.58). In patients whose primary cause of uremia was diabetic nephropathy, the score for PF was significantly lower than that in patients without diabetic nephropathy (60.59 ± 20.40 vs. 67.25 ± 29.62, *p < *.05). No significant differences in the remaining dimensions were observed **(**Table 5).

**Table 5.** Influencing factors on QOL (*p* values).

	Educational background	Medical insurance	Marriage	Residual renal function	Primary disease	Simple hemodialysis
PCS						
PF	0.990	0.897	0.526	0.678	**0.039**	0.776
PR	0.732	0.163	0.242	0.299	0.291	0.895
BP	0.376	0.919	0.341	0.196	0.330	0.165
GC	0.170	0.848	0.337	0.923	0.340	0.810
MCS						
VT	0.052	0.832	0.630	0.014	0.079	0.050
SF	0.402	0.914	0.812	0.939	0.110	0.317
RE	0.825	0.655	0.637	0.283	0.914	0.484
MH	**0.049**	0.973	0.417	0.063	0.946	0.574
Total score	0.267	0.887	0.403	0.214	0.151	0.208

*p* < .05 indicates statistical difference between groups within each influencing factor (marked in bold).

## Discussion

QOL was defined in this study as a person’s feelings regarding their living status with reference to their own objectives, anticipation and expectations under his/her own cultural and value systems. Medical care is improving and the long-term motility rate in blood dialysis patients is increasing. However, the influence of long-term illness and treatment on a patient’s physical, mental and social activities should not be ignored. Therefore, QOL in patients on maintenance hemodialysis has become a reliable index for the comprehensive evaluation of blood hemodialysis [2].

As a widely approved tool for testing and evaluating QOL, the SF-36 scale has been universally used to evaluate patients’ QOL [3]. According to previous research, blood dialysis patients treated for 1–2 years show a gradual improvement in QOL, which reaches a peak after 2–4 years of dialysis. In the present study, the total SF-36 score was 107.55 ± 14.50 in maintenance hemodialysis patients, which was higher than that in Guangzhou, China (total score: 74.8 ± 10.2) [4] and other areas in China [5]. These results may be due to the fact that the sample consisted of patients receiving blood dialysis for more than 2 years, had an overall understanding of their illnesses, the therapeutic measures undertaken, and they accepted the fact that they were sick. In addition, as patients with serious complications were excluded from this study, only cases with a stable illness were selected, and thus obtained higher QOL scores.

In the present study, female patients on hemodialysis generally had lower QOL scores compared with male patients, especially in the PF, RP and BP dimensions. These results are in line with the findings published by Peng YS [6]. This indicated that both male and female patients receiving hemodialysis for more than 2 years had accepted this treatment mode emotionally and spiritually. The differences in PF and RP between male and female patients were significant, and the reasons for these differences may be due to differences in physiology, psychology and self-requirement between males and females [7].

Furthermore, there were also differences in QOL between hemodialysis patients of different age groups. As shown in this study, the total QOL score decreased gradually with increasing age. In addition, the scores for PF, physical strength and SF also decreased gradually with increasing age. These results are consistent with those of previous studies. With increasing age, physical strength, energy and self-care ability decline continuously. The older the patients are, the weaker their physical functions will be and complications are more likely to occur. These complications in turn increase pain, and their physical functions and energy decline further. In addition, older age implies poorer ability regarding participation in social activities. Even when an elderly person’s body is in good condition, he/she is unlikely to reenter society. The hemodialysis patient’s ability to reenter society is poorer, [8] which also lowers his/her survival quality.

We found that, on the whole, educational background and source of funding did not have a significant influence on patient QOL. However, with regard to mental health, patients with a higher educational background obtained a significantly higher score than patients with a lower educational background. This may be because the former has a greater understanding of the value and importance of the treatment. In addition, patients with health insurance and those paying for treatment themselves showed no significant difference in QOL. Considering that the self-supporting patients were a minor proportion of the sample, the results did not show greater QOL in patients with health insurance.

Marital status was found to have no obvious influence on QOL. These results differ from the majority of previous studies, [9,10] and may be due to the fact that the patients in this study were almost all married. A few single patients are unlikely to indicate the influence of marital status on QOL in maintenance hemodialysis patients.

Residual urine favors the excretion of water and sodium, hydrogen and potassium ions, but also the elimination of middle-molecule toxins [11]. In this study, it was found that residual urine did not influence patient QOL. However, patients with residual urine had better energy than those without residual urine. According to our analysis, all patients receiving dialysis for more than 2 years did not have much renal function left. The patients with residual urine only had a urine volume of 100–200 mL/24 h. Thus, residual urine did not influence patient QOL.

According to previous reports, QOL in diabetic patients is markedly lower than that in non-diabetic patients [12]. In this study, diabetic patients had markedly lower scores in PF than non-diabetic patients. This might be because diabetes may lead to loss of function in multiple organs. In particular, diabetic patients receiving dialysis often have malfunctioning organs besides the kidney, and dialysis cannot reduce the damage in these other organs.

In conclusion, the QOL of patients receiving dialysis for more than 2 years was higher in Shanxi, compared with the QOL of patients from Guangzhou province, and other areas in China [4,5]. Gender and age were major factors affecting patient QOL, while educational background and residual urine may partially affect patient survival quality. Health insurance, marital status and mode of dialysis had little influence on patient QOL. It should be noted that in patients receiving dialysis for more than 2 years, few factors improved their QOL. Therefore, the treatment of hemodialysis patients in the first 2 years is very important in improving QOL and life satisfaction. The results of this study also provide evidence for the health authorities and medical workers to formulate comprehensive and effective therapeutic regimens.
